# Online Sexual Risk Behaviors in Adolescents: Roles of Family Relationships, Impulsivity, and Attention-Deficit/Hyperactivity Disorder

**DOI:** 10.3390/children11101199

**Published:** 2024-09-30

**Authors:** Wen-Jiun Chou, Tai-Ling Liu, Ray C. Hsiao, Cheng-Fang Yen

**Affiliations:** 1 Department of Child and Adolescent Psychiatry, Chang Gung Memorial Hospital, Kaohsiung Medical Center, Kaohsiung 83341, Taiwan; wjchou@cgmh.org.tw; 2 College of Medicine, Chang Gung University, Taoyuan City 33302, Taiwan; 3 Department of Psychiatry, Kaohsiung Medical University Hospital, Kaohsiung 80708, Taiwan; 4 Department of Psychiatry, School of Medicine, College of Medicine, Kaohsiung Medical University, Kaohsiung 80708, Taiwan; 5 Department of Psychiatry and Behavioral Sciences, University of Washington School of Medicine, Seattle, WA 98195, USA; rhsiao@u.washington.edu; 6 Department of Psychiatry, Children’s Hospital and Regional Medical Center, Seattle, WA 98105, USA; 7 College of Professional Studies, National Pingtung University of Science and Technology, Pingtung 91201, Taiwan

**Keywords:** online risk behavior, sex, attention-deficit/hyperactivity disorder, family relationship, impulsivity, psychological well-being

## Abstract

Purpose: This study examined the associations of passive and active online sexual risk behaviors with perceived family relationships, impulsivity, and attention-deficit/hyperactivity disorder (ADHD). Participants and methods: A total of 176 adolescents with ADHD and 173 adolescents without ADHD participated in this study. The participants’ parents rated their parenting style on the Parental Bonding Instrument. The adolescents self-reported their lifelong experiences of passive and active online sexual risk behaviors, perceived family relationship quality on the family domain of the Taiwanese Quality of Life Questionnaire for Adolescents, and three domains of impulsivity on the Barratt Impulsiveness Scale version 11. Multivariable logistic regression was used to examine the associations of online sexual risk behaviors with perceived family relationships, impulsivity, and ADHD. Results: Overall, 114 participants (32.7%) reported passive forms of online sexual risk behaviors, and 49 (14.0%) reported active online sexual risk behaviors. Lack of foresight and self-control was significantly associated with passive online sexual risk behaviors (*p* = 0.003). Good family relationship was significantly associated with a decreased risk of active online sexual risk behaviors (*p* = 0.011), whereas seeking novelty and making decisions hastily was significantly associated with an increased risk of active online sexual risk behaviors (*p* = 0.048). ADHD diagnosis and inability to plan were not significantly associated with online sexual risk behaviors (*p* > 0.05). Conclusion: A high proportion of Taiwanese adolescents reported exhibiting online sexual risk behaviors. The factors related to the manifestation of these behaviors should be considered when designing relevant intervention programs.

## 1. Introduction

An increasing number of adolescents are using the Internet to obtain new ideas, social contact and support, and health-related information [1]. However, excessive Internet use negatively affects the sleep, attention, and learning capabilities of individuals and is associated with increased incidence of obesity and mental health problems [1,2]. For example, studies have found that nighttime social media use was associated with a poorer sleep quality, which in turn associated with more aggressive behaviors [3]. Online sexual risk behaviors—such as unwanted online sexual exposure, sexting, and exposure to pornography—are common in adolescents [4]. Unwanted online sexual exposure is defined as a situation in which a person is induced by others to talk about sex, share personal sexual information, or involuntarily engage in sexual behaviors online [5,6]. Online sexual victimization is a wide range of behaviors that are unwanted by the person in an online context, such as exposure to sexual material, sexual pressure and online grooming, and requests to engage in sexual activity or talk or to provide personal sexual information [7,8]. A meta-analysis discovered that 20.3% of adolescents had experienced unwanted sexual exposure online and that 11.5% had received unwanted sexual advances online [9]. An Italian study found that 60% of adolescents aged between 12–14 years reported at least one form of online sexual victimization [7]. Unwanted online sexual exposure can cause distress in adolescents, especially younger adolescents and those coerced to make offline contact [5].

Sexting is defined as the electronic transmission of nude or seminude images or sexually explicit text messages [10]. Approximately 12% of adolescents aged 10–19 years have reportedly sent a sexual photograph of themselves to someone online [11]. Studies have discovered that individuals who send or receive sexual messages often display comorbid internalizing problematic behaviors, addictive substance use behavior, and impulsive behavior [12,13,14]. Adolescents are also commonly exposed to pornography and sexually explicit websites [15]. A meta-analysis revealed that young people who have accessed sexually explicit websites are more likely to engage in condomless sex [16]. Moreover, regular watchers of pornography are more likely to exhibit coercive sexual behaviors and engage in sexual abuse [16,17]. Studies have underscored the importance of implementing interventions for reducing the involvement of adolescents in online sexual risk behaviors. The factors related to online sexual risk behaviors should be identified and addressed in effective intervention programs.

According to ecological system theory [18], individual and environmental factors contribute to adolescents’ online sexual risk behaviors. One such individual risk factor is attention-deficit/hyperactivity disorder (ADHD). Studies have found that the individuals with ADHD have high tendencies of boredom and intolerance of delayed reward [19,20]. They may receive rapid reward from the Internet to avoid boredom. Adolescents with ADHD may have difficulties in inhibition of impulsivity [21,22], which may increase their risk of problematic Internet use. Studies have demonstrated that children and adolescents with ADHD are more likely to engage in sexual risk behaviors—such as contracting sexually transmitted diseases and having early sexual intercourse, multiple sexual partners, and sex outside of regular relationships—compared with individuals without ADHD [23,24,25]. ADHD is also a risk factor for problematic Internet use in adolescents [26]. However, one study reported that the likelihood of an adolescent with ADHD engaging in sexting was the same as that for the general population [27]. Given that the results of previous studies on involvement in online sexual risk behaviors in adolescents with ADHD are in mixed [23,24,25,26,27], further investigations are warranted to determine whether adolescents with ADHD are more likely to exhibit online sexual risk behaviors than those without ADHD.

One of the environmental factors that protect an adolescent from mental health problems is good family relationships [28]. Good family relationships were found to protect female adolescents from online sexual abuse [29], whereas family dysfunction increased the risk of online sexual victimization in adolescents [30]. Although it has been found that parental efforts to control adolescents’ Internet use for guarding the safety of adolescents is ineffective [31], family members can provide adolescents with family interactions and activities that may reduce their necessity to look for online interactions and activities [32]. Good family relationships also provide parents and adolescents the opportunity to discuss sexual issues; adolescents can explore sex appropriately in real life [33]. However, the associations between the quality of family relationships and various online sexual risk behaviors, such as engaging in sexting, have not yet been examined.

Another individual factor that may correlate with online sexual risk behaviors in adolescents is impulsivity. Impulsivity is characterized by nonreflective responses, uncontrollable desires, repetitive behaviors, and the underestimation of harm to obtain pleasure and gratification [34]. High impulsivity was also shown to elevate the risk of problematic Internet use [35]. Highly impulsive adolescents may ignore the potential dangers of the online world and interact with unknown people, thereby increasing their risk of passive online sexual risk behaviors. Highly impulsive adolescents may also send sexual messages or visit sexually explicit websites without giving these behaviors much thought. However, impulse is a multidimensional concept. For example, according to Barratt [36], impulsivity contains three aspects, including the inability to plan, a lack of foresight and self-control, and a proclivity toward seeking novelty and making decisions hastily. Further studies are required to clarify the association between various aspects of impulsivity and online sexual risk behaviors.

This study examined the associations between passive (including being invited to talk about sex-related topics and engage in sexual behaviors, being pressured to share personal sexual information, receiving messages containing sexual content, pictures or videos with sexual connotations, or seminude or fully nude pictures or videos, and inadvertently viewing sexually explicit information or videos) and active online sexual risk behaviors (including sending messages with sexual content to others, pictures or videos with sexual connotations to others, or seminude or fully nude pictures or videos to others, and searching for sexually explicit information or watched pornography) in adolescents and the adolescents’ perceived family relationships, impulsivity, and ADHD status. We have three hypotheses. First, we hypothesized that higher-quality family relationships are associated with a lower risk of online sexual risk behaviors. Second, we hypothesize that various domains of impulsivity have different associations with the risk of online sexual risk behaviors. Third, we hypothesized that adolescents with ADHD are more likely to exhibit online sexual risk behaviors than are those without ADHD.

## 2. Methods

### 2.1. Participants and Procedure

This study recruited adolescents with ADHD and typically developing (TD) adolescents without ADHD between September 2022 and July 2023. The adolescents who had a diagnosis of ADHD and aged between 11 to 18 years were recruited from six child psychiatry outpatient clinics in two hospitals in Taiwan. ADHD was diagnosed by applying multiple data sources, including (a) an interview conducted by a certified child psychiatrist in accordance with the *Diagnostic and Statistical Manual of Mental Disorders, Fifth Edition* [37], (b) clinical observation of the adolescent’s behaviors, (c) the results of reviewing the adolescent’s medical records, and (d) the adolescent’s history of behaviors and attention provided by the parents. Since these adolescents with ADHD received pharmacological treatment or psychotherapy currently and their ADHD symptoms might be mitigated, this study did not use the measures for the severity of ADHD symptoms to make the diagnosis. A total of 190 adolescents with ADHD who met the inclusion criteria were identified. Three child psychiatrists conducted clinical interviews with the adolescents with ADHD and their parents or guardians and reviewed adolescents’ medical charts to determine whether the adolescents had comorbid intellectual disability or other psychiatric disorders. If the adolescents had any comorbid psychiatric disorder that impeded their ability to understand the study’s aims or completing the study questionnaire, they were not invited to participate in this study. In total, 176 adolescents with ADHD were eligible and agreed to participate in the study.

TD adolescents (without ADHD) were recruited through online advertising. TD adolescents were included in this study if (1) their age was in the range 11 to 18 years and (2) they did not have ADHD, intellectual disability, or other psychiatric disorders that impeded their ability to understand the study’s aims and completing the study questionnaire. The child psychiatrists interviewed the adolescents and their parents or guardians to confirm that the adolescent was eligible for inclusion. In total, 173 TD adolescents agreed to participate in this study. Both the adolescents and their parents in the ADHD and TD groups provided written informed consent. The protocol of the present study was approved by the Institutional Review Boards of Kaohsiung Medical University Hospital (IRB protocol number = KMUHIRB-SV (I)-20200091 and date of approval = 5 August 2022) and Chang Gung Memorial Hospital, Kaohsiung Medical Center (IRB protocol number = 202101964A3C502 and date of approval = 18 August 2022).

### 2.2. Measures

#### 2.2.1. Passive and Active Types of Online Sexual Risk Behaviors

This study reviewed previous studies investigating online sexual risk behaviors and selected appropriate questions to investigate the various dimensions of online sexual risk behaviors. We assessed the participants’ lifetime experiences with seven passive types and four active types of online sexual risk behaviors. The seven items used to assess the participants’ passive types of online sexual risk behaviors were adopted from other studies [15,27,38] and inquired about whether the participant had been invited to talk about sex-related topics, been pressured to share personal sexual information, been invited to engage in sexual behaviors, received messages containing sexual content, received pictures or videos with sexual connotations, received seminude or fully nude pictures or videos, and inadvertently viewed sexually explicit information or videos. The participants who answered “yes” to any item were considered to have exhibited passive types of online sexual risk behaviors. Four items were employed to assess the participants’ active types of online sexual risk behaviors were adopted from other studies [15,27] and inquired about whether the participant had sent messages with sexual content to others, sent pictures or videos with sexual connotations to others, sent seminude or fully nude pictures or videos to others, and searched for sexually explicit information or watched pornography. The participants who answered “yes” to any item were considered to have exhibited active types of online sexual risk behaviors.

#### 2.2.2. Family Relationship Quality

The level of adolescents’ satisfaction in family relationships was assessed using the seven-item family relationship subscale of the Taiwanese Quality of Life Questionnaire for Adolescents (TQOLQA; example item, “Are you satisfied with the help and support you receive from your family?”) [39]. Each item was rated on a 5-point Likert scale ranging from 1 (not at all) to 5 (very much). A high total score indicated high satisfaction in family relationships. The Cronbach’s alpha for the family relationship subscale of the TQOLQA was 0.89 in the present study.

#### 2.2.3. Impulsivity

The self-reported level of the adolescents’ impulsivity was evaluated using the Barratt Impulsiveness Scale version 11—Taiwan (BIS-11-TW) [34,40]. The 25-item BIS-11-TW measures three aspects of impulsivity, including the inability to plan (for example, “I do things without thinking”), a lack of foresight and self-control (for example, “I buy things on impulse”), and a proclivity toward seeking novelty and making decisions hastily (for example, “I make up my mind quickly”). Each item was measured on a 4-point scale ranging from 1 (never or rarely) to 4 (almost always or always). A higher total score of the subscale indicates a higher level of impulsivity. The BIS-11-TW has acceptable psychometric properties [34]. The Cronbach’s alpha was 0.88 in the present study.

### 2.3. Data Analysis

This study used IBM SPSS Statistics version 24.0 (IBM Corporation, Armonk, NY, USA) to analyze the data. Chi-square and *t* tests were used to compare the demographics, perceived family relationships, impulsivity on the BIS-11-TW, and passive and active types of online sexual risk behaviors between the participants of the ADHD and TD groups. Absolute values of kurtosis < 7 and skewness < 3 of the continuous variables indicate normal distribution [41]. The results did not reveal any significant deviation. The correlations between variables were examined using Pearson’s correlation (for continuous variables) or Spearman’s correlation (for dichotomous variables). The correlation coefficient below 0.3, 0.3 to 0.5, and 0.5 above indicates a small, medium, and large strength of association, respectively. Multivariate logistic regression was used to examine the associations of demographic characteristics, ADHD, quality of family relationships, and impulsivity with the experiences of passive and active types of online sexual risk behaviors. All independent variables were included simultaneously. The results are presented as odds ratios and 95% confidence intervals. A *p* value < 0.05 was considered to indicate statistical significance. We also used the standard criteria proposed by Baron and Kenny [42] to examine the moderating effect of family relationships on the association of impulsivity with online sexual risk behaviors.

## 3. Results

Table 1 presents a comparison of the demographic characteristics, family relationship quality, and impulsivity of the ADHD and TD groups. The TD group had a higher family relationship score on the TQOLQA than did the ADHD group (*p* = 0.015). The ADHD group had higher scores for inability to plan (*p* < 0.001) and lack of foresight and self-control on the BIS-11-TW (*p* = 0.003) compared with the TD group. The two groups did not differ significantly in their scores for seeking novelty or making decisions hastily on the BIS-11-TW (*p* > 0.05). A total of 114 participants (32.7%) reported passive types of online sexual risk behaviors, whereas 49 participants (14.0%) reported active types of online sexual risk behaviors. The two groups did not differ significantly in the proportion of adolescents exhibiting passive or active online sexual risk behaviors (*p* > 0.05).

Table 2 presents the correlations between the variables. The diagnosis of ADHD negatively correlated with family relationships and positively correlated with inability to plan and lack of foresight and self-control. Girls reported higher family relationship quality than did boys. Family relationship negatively correlated with the three domains of impulsivity on the BIS-11-TW. The strengths of these association were small. The three domains of impulsivity on the BIS-11-TW positively correlated with each other with a small to medium strength of association. Age, lack of foresight and self-control, and seeking novelty and making decisions hastily positively correlated with passive online sexual risk behaviors. Being boys, age, and the three domains of impulsivity positively correlated with active online sexual risk behaviors, whereas family relationship negatively correlated with active online sexual risk behaviors. The diagnosis of ADHD did not significantly correlate with passive or active online sexual risk behaviors.

Table 3 presents the results for the associations of online sexual risk behaviors with family relationship quality, impulsivity, and ADHD. After the effects of sex and age had been adjusted for, the score for lack of foresight and self-control on the BIS-11-TW was significantly associated with increased likelihood of passive online sexual risk behaviors (*p* = 0.003). The family relationship score on the TQOLQA was significantly associated with decreased likelihood of active online sexual risk behaviors (*p* = 0.011), whereas the score for seeking novelty and making decisions hastily on the BIS-11-TW was significantly associated with increased likelihood of active online sexual risk behaviors (*p* = 0.048). The diagnosis of ADHD and score for inability to plan on the BIS-11-TW were not significantly associated with passive or active online sexual risk behaviors (*p* > 0.05). the interaction between family relationships and seeking novelty and making decisions hastily was further entered in the logistic regression analysis. The interaction variable was not significantly associated with active online sexual risk behaviors, indicating that family relationships did not moderate the association between seeking novelty and making decisions hastily and active online sexual risk behaviors.

## 4. Discussion

A high proportion of the Taiwanese adolescents surveyed in this study reported online sexual risk behaviors, with 32.7% having exhibited the passive type and 14.0% having exhibited the active type of online sexual risk behaviors. This study assessed any passive type online sexual risk behaviors, and the rate was higher than the results of previous studies on individual type of online sexual risk behaviors (20.3% having unwanted sexual exposure online and 11.5% having received unwanted sexual advances online [9]). Alternatively, the rate of of adolescents with active type online sexual risk behaviors was similar to the results of a previous study (12% sending a sexual photograph of themselves to someone online [11]).

Poor-quality family relationships were significantly associated with active online sexual risk behaviors. According to ecological system theory [18], both family and the Cyberworld are the microsystem that the adolescents interact with closely. Although a person seeks approval from people outside the family during adolescence, the family still provides basic support for the adolescent’s life and emotional well-being [43]. Alternatively, the Cyberworld offers adolescents a wide variety of interpersonal interactions, entertainment, and values. In such complex individual-microsystem interactions, good family relationships may mitigate the risk of adolescents engaging in active online sexual risk behaviors in the following ways. First, good family relationships can provide adolescents with the interpersonal interactions and activities they require, reducing their desire to seek interpersonal relationships and fun on the Internet [32], which in turn will decrease their involvement in active online sexual risk behaviors. Second, if an adolescent has a good relationship with their family, they are likely to allow appropriate parental supervision of their Internet use, which can reduce the incidence of active online sexual risk behaviors [44]. Third, when family relationships are good, parents and children are more likely to discuss sexual issues. Adolescents raised in such families can explore sex appropriately in real life [33], leading to a lower risk of active online sexual risk behaviors.

The present study uncovered a significant association between impulsivity and online sexual risk behaviors. In terms of biological brain functioning, dopamine functioning is an essential mechanism underlying impulsivity, sexual exploration, and problematic Internet use [45,46,47]. The complex interactions among the three behaviors are inherently biological in nature. Moreover, lack of foresight and self-control was positively associated with passive online sexual risk behaviors. Interpersonal interactions in the online world are anonymous, and adolescents are likely to receive probing contact from Internet users with whom they are unfamiliar. A lack of foresight and self-control on the part of adolescents can easily result in them being exposed to sexual invitations or sexting from Internet users. This study also demonstrated that seeking novelty and making decisions hastily were significantly associated with active online sexual risk behaviors. Given that sexual exploration is a salient developmental feature associated with adolescence [43], adolescents who seek novelty and make decisions hastily may be motivated by an intrinsic need to send sexual invitations to or engage in sexting with others in the online world without giving much thought to the appropriateness of such behavior. Finally, the present study found that the inability to plan was not significantly associated with passive or active online sexual risk behaviors. The results indicate that most online sexual risk behaviors are unplanned behaviors, so the ability to devise and execute a plan is not expected to be significantly related to online sexual risk behaviors.

Given that sexual risk behaviors and problematic Internet use are more likely to occur in individuals with ADHD [23,24,25,26], researchers have hypothesized that adolescents with ADHD are more likely to demonstrate online sexual risk behaviors. However, the present study did not find significant differences in the risks of online sexual risk behaviors between adolescents with versus without ADHD. The findings indicate that interventions for reducing the risk of online sexual risk behaviors should be implemented among all adolescents in the general population.

On the basis of the findings of this study, the following recommendations can be made regarding how the physical, mental, and legal distress caused by adolescents’ online sexual risk behaviors can be mitigated. First, health-care professionals should regularly assess online sexual risk behaviors in adolescents. Adolescents with or without an ADHD diagnosis should be evaluated. Health-care professionals should educate the public about online sexual risk behaviors and the negative consequences it can do to adolescents. They should also clearly delineate the disadvantages of online sexual risk behaviors and implement strategies to prevent these behaviors among adolescents. Second, health-care professionals should help families cultivate good relationships to reduce the likelihood of adolescents’ exhibiting to online sexual risk behaviors. Health-care professionals should understand the impulsive nature of adolescents and help them develop the ability to think ahead, exercise self-control, and make risk-adjusted decisions to reduce the likelihood they will exhibit online sexual risk behaviors.

The present study has some limitations. First, this study recruited adolescents with ADHD from outpatient clinics, and all adolescents in the ADHD group were currently receiving medication or psychological interventions for ADHD. Further study is needed to ascertain the generalizability of this study to adolescents with ADHD who do not receive medical help. This study recruited TD adolescents through online advertisements. Further study is needed to ascertain the generalizability of this study to adolescents who are not recruited through online advertisements. Further, the difference in recruitment methods of adolescents with ADHD and TD adolescents could have introduced sampling bias. Second, given the cross-sectional design of the present study, the temporal associations between online sexual risk behaviors and other variables could not be determined. Further follow-up study is needed to examine the the temporal associations between online sexual risk behaviors, family relationships, and impulsivity. Third, given that participants’ self-reported data were collected in this study, the results are prone to single-rater and recall biases. Future studies are warranted to examine potential social desirability bias. In addition, obtaining information from multiple sources may verify the accuracy of the findings of this study. Fourth, this study adopted multiple items from previous studies to assess participants’ passive and active types of online sexual risk behaviors [15,27,38]; however, the reliability and validity of the scale warrants further study.

## 5. Conclusions

The results of this study indicate that a high proportion of Taiwanese adolescents have exhibited online sexual risk behaviors. Health-care professionals should regularly assess online sexual risk behaviors among adolescents, spread awareness about their risks, and implement strategies to prevent these behaviors. The quality of family relationships, a lack of foresight and self-control, seeking novelty, and making decisions hastily were significantly associated with online sexual risk behaviors. Health-care professionals should assess adolescents’ family relationships and impulsivity and develop corresponding interventions to reduce the risk of online sexual risk behaviors.

## Figures and Tables

**Table 1:** Demographics, quality of family relationships, impulsivity, and online sexual risk behaviors between adolescents with ADHD and adolescents with TD (*n* = 349).

	Total (*n* = 349)	Typical Development (*n* = 173)	ADHD (*n* = 176)	*t* or χ^2^	*p*
Age (years), mean (SD)	13.7 (2.1)	13.7 (2.1)	13.7 (2.1)	−0.149	0.881
Sex, *n* (%)					
Female	64 (18.3)	32 (18.5)	32 (18.2)	0.006	0.939
Male	285 (81.7)	141 (81.5)	144 (81.8)		
Perceived family relationship quality on the TQOLQA, mean (SD)	27.5 (5.3)	28.2 (4.9)	26.8 (5.6)	2.439	0.015
Impulsivity on the BIS-11-TW, mean (SD)					
Inability to plan	21.4 (4.2)	20.4 (4.0)	22.4 (4.2)	−4.620	<0.001
Lack of foresight and self-control	23.9 (4.7)	23.2 (4.4)	24.6 (4.8)	−2.993	0.003
Seeking novelty and making decisions hastily	15.2 (3.3)	15.0 (2.8)	15.5 (3.7)	−1.359	0.175
Passive type of online sexual risk behaviors	114 (32.7)	57 (32.9)	57 (32.4)	0.013	0.911
Active type of online sexual risk behaviors	49 (14.0)	25 (14.5)	24 (13.6)	0.048	0.827

ADHD: attention-deficit/hyperactivity disorder; TQOLQA: Taiwanese Quality of Life Questionnaire for Adolescents; BIS-11-TW: Barratt Impulsiveness Scale version 11—Taiwan.

**Table 2:** Correlation matrix for the variables.

	1	2	3	4	5	6	7	8	9
1. Sex	–								
2. Age	−0.015	–							
3. ADHD	0.004	0.007	–						
4. Family relationships	−0.111 *	−0.066	−0.122 *	–					
5. Inability to plan	0.060	−0.089	0.269 ***	−0.268 ***	–				
6. Lack of foresight and self-control	0.081	0.077	0.156 **	−0.309 ***	0.476 ***	–			
7. Seeking novelty and making decisions hastily	0.095	−0.004	0.077	−0.116 *	0.137 *	0.470 ***	–		
8. Passive online sexual risk behaviors	0.093	0.158 **	0.006	−0.073	0.010	0.236 ***	0.160 **	–	
9. Active online sexual risk behaviors	0.149 **	0.249 ***	−0.012	−0.204 ***	−0.018	0.177 **	0.148 **	0.369 ***	–

* *p* < 0.05; ** *p* < 0.01; *** *p* < 0.001.

**Table 3:** Associations of online sexual risk behaviors with quality of family relationships, impulsivity, and ADHD: Multivariable logistic regression analysis.

Variable	Passive Online Sexual Risk Behaviors		Active Online Sexual Risk Behaviors			
			Model I		Model II	
	OR (95% CI)	*p*	OR (95% CI)	*p*	OR (95% CI)	*p*
Sex ^a^	1.613 (0.835–3.116)	0.155	5.687 (1.279–25.281)	0.022	5.745 (1.289–25.598)	0.022
Age	1.163 (1.040–1.302)	0.008	1.426 (1.213–1.677)	<0.001	1.427 (1.213–1.678)	<0.001
ADHD ^b^	0.851 (0.524–1.383)	0.515	0.772 (0.389–1.533)	0.459	0.765 (0.383–1.525)	0.446
Family relationships	1.000 (0.953–1.048)	0.987	0.917 (0.858–0.980)	0.011	0.957 (0.683–1.343)	0.801
Inability to plan	0.967 (0.903–1.035)	0.336	0.926 (0.840–1.021)	0.123	0.926 (0.839–1.021)	0.122
Lack of foresight and self-control	1.106 (1.034–1.183)	0.003	1.059 (0.963–1.163)	0.236	1.061 (0.964–1.167)	0.227
Seeking novelty and making decisions hastily	1.065 (0.980–1.158)	0.136	1.127 (1.001–1.269)	0.048	1.206 (0.711–2.045)	0.487
Family relationships x Seeking novelty and making decisions hastily					0.997 (0.978–1.017)	0.798

^a^: girls as the reference; ^b^: TD adolescents as the reference.

## Data Availability

The data are available upon reasonable request to the corresponding authors. The data are not publicly available due to privacy.
